# Cognitive Trajectories in Comorbid Dementia With Schizophrenia or Bipolar Disorder: The South London and Maudsley NHS Foundation Trust Biomedical Research Centre (SLaM BRC) Case Register

**DOI:** 10.1016/j.jagp.2020.10.018

**Published:** 2021-06

**Authors:** Rebecca Bendayan, Aurelie Mascio, Robert Stewart, Angus Roberts, Richard J. Dobson

**Affiliations:** 1 Department of Biostatistics and Health Informatics, Institute of Psychiatry, Psychology and Neuroscience (RB, AM, AR, RJD), King's College London, London, United Kingdom; 2 NIHR Biomedical Research Centre at South London and Maudsley NHS Foundation Trust (RB, AR, RJD), London, United Kingdom; 3 Department of Psychological Medicine, Institute of Psychiatry, Psychology and Neuroscience (RS), King's College London, London, United Kingdom; 4 Institute of Health Informatics (RJD), University College London, London, United Kingdom

**Keywords:** Dementia, severe mental illness, cognitive decline, electronic health records

## Abstract

**Objectives:**

We aimed to compare trajectories of cognitive performance in individuals diagnosed with dementia with and without severe mental illness (SMI).

**Design:**

Retrospective cohort study.

**Setting:**

We used data from a large longitudinal mental healthcare case register, the Clinical Record Interactive Search (CRIS), at the South London and Maudsley NHS Foundation Trust (SLaM) which provides mental health services to four south London boroughs.

**Participants:**

Our sample (N = 4718) consisted of any individual who had a primary or secondary diagnosis of dementia from 2007 to 2018, was 50 years old or over at first diagnosis of dementia and had at least 3 recorded Mini-Mental State Examination (MMSE) scores.

**Measurements:**

Cognitive performance was measured using MMSE. Linear mixed models were fitted to explore whether MMSE trajectories differed between individuals with or without prior/current SMI diagnoses. Models were adjusted by socio-demographics, cardiovascular risk, smoking, and medication.

**Results and conclusions:**

Our results showed differences in the rate of change, where individuals with comorbid SMI had a faster decline when compared with those that have dementia without comorbid SMI. However, this association was partially attenuated when adjusted by socio-demographics, smoking and cardiovascular risk factors; and more substantially attenuated when medication was included in models. Additional analyses showed that this accelerated decline might be more evident in individuals with bipolar disorders. Future research to detangle the potential biological underlying mechanisms of these associations is needed.

## Highlights

• We compared the cognitive trajectories in individuals with dementia with and without comorbid severe mental illness using data from one of the largest mental health care providers in Europe.
• Our results showed that individuals with comorbid SMI were more likely to have a faster decline in their MMSE scores compared with those that had dementia without this comorbidity. However, this association was attenuated when considering socio-demographics, smoking and cardiovascular risk factors and medication.
• Our findings highlight the potential risk for an accelerated cognitive decline in this group of individuals, specially for those with bipolar disorders, and the need to further investigate the role of potential shared mechanisms.

## Introduction

Most research on cognitive decline in individuals with dementia does not include individuals with comorbid severe mental illness (SMI; e.g., schizophrenia and bipolar disorder). This is because individuals with comorbidities in general, and mental health illness in particular, are traditionally excluded from large population studies or their numbers are very small. In recent decades, there has been a growing interest to include these under-represented subpopulations. As a result, most studies have explored cognitive performance in individuals diagnosed with SMI but not dementia. Most of these studies have explored cross-sectional differences between individuals with bipolar disorders and schizophrenia^1^ and their results tend to show similar cognitive profiles for both.^2^^,^^3^ However, some studies have suggested that differences might be arise when exploring this association in longitudinal settings.^4^

To date, most longitudinal studies have not found differences between cognitive trajectories in individuals with schizophrenia and bipolar disorders.^5^, ^6^, ^7^ For example, Gildengers et al.^5^ compared the trajectories of cognitive performance in outpatients with bipolar disorder with healthy controls over 2 years and they found that although individuals with SMI had overall lower cognitive performance, their rates of change did not differ. Other studies also compared cognitive performance and trajectories between individuals with bipolar disorder and schizophrenia and found no relevant differences.^6^^,^^7^ A recent review highlighted that most of longitudinal studies have small sample sizes and they might be underpowered to detect subtle cognitive changes over time or to reveal differences between schizophrenia and bipolar disorders.^1^ Therefore, further research with larger sample sizes and longer follow ups are needed.

It has been suggested that the short term cognitive impairments associated with acute exacerbations of mental disorders do not result in an accelerated cognitive decline,^5^ other studies have suggested that psychosis could be a risk factor for the development of dementia^8^^,^^9^ and subsequent cognitive decline in individuals with other comorbidities such as Parkinson disease.^10^ Potential shared biological mechanisms could be associated with alterations of hypothalamic–pituitary–adrenal axis, mitochondrial dysfunction/oxidative stress, glutamatergic abnormalities found in bipolar disorders and dementia^11^^,^^12^ and/or with common cardiovascular risk factors which are independently associated with antipsychotic medication intake^12^ and with dementia risk.^8^^,^^9^ However, some authors do not find that these play a role in the increased risk for dementia in individuals with bipolar disorders.^13^ Less is known about the potential shared links between schizophrenia and dementia. Schizophrenia is considered a neurodevelopmental disorder with its distinct underlying biological mechanisms such as aberrant pruning of synapses^14^ and although previous research has discussed potential shared mechanisms with dementia,^15^, ^16^, ^17^ evidence remains scarce and inconclusive.

Given this context, there is an ongoing debate on the nature of the progression of cognitive changes in schizophrenia and bipolar disorders and it is yet not clear whether these are associated with a neurodevelopmental or neurodegenerative process such as dementia. Understanding the underlying biological mechanisms between SMI such as schizophrenia or bipolar disorders and dementia could contribute to improve healthcare and reveal potential new therapeutic targets. Therefore, there is a need for research to disentangle the complex inter-relationships between SMI and dementia, especially given that individuals with this comorbidity pattern are at increased risk of psychiatric hospitalizations and higher levels of health care utilization^12^^,^^18^ and may share common determinants such as health behaviors or cardiovascular risk factors.^18^^,^^19^ The main aim of this study was to compare trajectories of cognitive performance in individuals diagnosed with dementia with and without SMI diagnosis using data from a large longitudinal mental healthcare case register, and to investigate associations with sociodemographic status, health behaviors, cardiovascular risk factors, and medication. In addition, we investigate whether different SMI diagnosis (schizophrenia and bipolar disorders) showed different cognitive trajectories when compared with individuals with only dementia.

## METHODS

### Setting and Sample

Data were extracted from the South London and Maudsley NHS Foundation Trust (SLaM) Clinical Record Interactive Search (CRIS). SLAM provides mental health services to a defined geographic catchment area of four south London boroughs (Lambeth, Southwark, Lewisham, and Croydon), with populations comparable overall with those of London as a whole in terms of age, gender, education and socio-economic status distributions.^20^ CRIS provides researcher access to deidentified data from SLaM's electronic health record which has been used across all services since 2006 and which has been substantially enhanced through a range of natural language processing (NLP) algorithms, as described in detail in an open-access catalogue (https://www.maudsleybrc.nihr.ac.uk/facilities/clinical-record-interactive-search-cris/cris-natural-language-processing/). The sample (N = 4718) consisted of any individual who had a primary or secondary diagnosis of dementia from 2007 to 2018, 50 years old or over at first diagnosis of dementia and at least three recorded MMSE scores, following Singer and Willet^21^ guideline. These individuals were similar for most of the relevant variables included in our study to those that were less than 50 years old at first diagnosis of dementia and those with at least one MMSE score but less than 3 MMSE measures (n = 10023). Within included individuals we found slightly higher percentages of Black Caribbean and married or cohabiting individuals and higher levels of education. Diagnoses are recorded in SLaM according to the International Classification of Mental and Behavioural Disorders-10 (ICD-10). Dementia was defined based on codes F00, F01, F02, F03, or F04; SMI included schizophrenia-spectrum disorders, bipolar affective disorders and maniac episodes (codes F20-29, F30-31).

### Variables

Cognitive performance. Cognitive performance was measured using extracted scores from the Mini-Mental State Examination (MMSE).^22^ The MMSE consists of 21 questions on orientation, immediate and delayed recall, naming, spelling, and simple arithmetic and constructional praxis. Higher scores indicate better cognitive performance. We used data from structured fields, and when these were not available, we extracted data from unstructured text fields, using NLP algorithms as in;^23^ 13% of scores were derived from structured fields, and 87% using NLP.

Covariates. Socio-demographic covariates included age at baseline (i.e., when first MMSE was recorded), sex, education, ethnicity, marital status, deprivation index and English as first language. Education was derived from records as a binary variable with two levels, primary or lower education and secondary or higher education (i.e., at least GSCE or equivalent), and extracted using NLP algorithms.^24^ Ethnicity was grouped into White, Black African, Black Caribbean, Indian, mixed or other. Marital status was grouped in two categories: married/cohabiting and single/separated/divorced/widowed. Neighborhood deprivation was measured at Lower Super Output Area level (a standard UK residence unit with a mean population size of 1,500 residents) using the address recorded current or most recent at the index date, linked to the Index of Multiple Deprivation (IMD) score for that neighborhood, extracted from 2011 national Census data (Department for Communities and Local Government, 2015). IMD scores were then categorized as deciles with lower deciles were indicative of higher deprivation. Smoking status extracted using an NLP algorithm^25^ and individuals were categorized as never, current, and former smokers. Cardiovascular risk factors such as obesity, diabetes and hypertension were combined in an index which was used to classify individuals as normal or at risk. Data was extracted using a combination of structured data when available and NLP techniques.^24^ Normal status was defined as a BMI between 18.5 and 25 (National, Clinical Guideline Centre UK, 2014), a systolic/diastolic blood pressure ratio up to 120/80 (National High Blood Pressure Education Program, 2003) and plasma glucose level lower than 5.5 (National Institute for Health and Care Excellence, 2012) Individuals were considered at cardiovascular risk when at least one of these indicators was not within clinically normal ranges.

Medication was extracted using a combination of structured and NLP derived data,^24^ identifying mentions or not of prescribed medications in three categories for this analysis: antipsychotic, antidepressant, and dementia-treatment agents (list provided in Supplemental Appendix 1).

### Statistical Analyses

Preliminary analyses were performed to describe the characteristics of the sample and compare these between individuals diagnosed with SMI or not. To examine group differences in cognitive trajectories and investigate associations with covariates, we used linear mixed models with random coefficients. The main advantage of using linear mixed models is that they allow us to account for between and within individual variability.^21^ Linear and quadratic unconditional models were examined. Chronological age measured in 6-month intervals as the time metric. Intraclass coefficients (ICC) for unconditional models were estimated as a measure of variation. ICCs of between 0.20 and 0.80 are suggestive of between- and within-individual variations (i.e., differences between individuals and change over time). In order to investigate the independence of the association of interest, we first ran an unadjusted model (model 1), then a model adjusted by socio-demographic factors (model 2), followed by additional cumulative adjustments for smoking (model 3), cardiovascular risk factors (model 4), and medication (model 5). The fit of the models was compared using the Bayesian Information Criterion (BIC), which is an index that combines information on a model's goodness of fit and parsimony. Lowest BIC is an indicator of better fit. Effect sizes for slopes were estimated using standardized regression coefficients.^26^

Missing values in some of our covariates was larger than 30% (ethnicity [n = 21, 0.44%], marital status [n = 51, 1.08%], IMD [n = 976, 20.68%], smoking status [n = 492, 10.42%], CVD risk [n = 1533, 32.49%] and first language [n = 1,995, 42.28%]), thus our models included a unknown category for these variables. Sensitivity analyses were performed to compare these models with cases with complete data for all covariates (n = 1,453). In addition, further sensitivity analyses were performed to ensure the robustness of our results considering attrition due to death or potential variance heterogeneity associated to age at diagnoses. In order to examine whether there could be any potential bias associated to the premature mortality in individuals with SMI we replicated our analyses restricting the sample to the individuals who were still alive at 2019 (n = 1766) and compared the results.

## Results

As can be seen in Table 1, individuals with dementia and SMI had slightly higher MMSE scores than those individuals with dementia alone [t(20826) = −8.71; p <0.001]. For all other variables exact Chi-square were performed. Individuals with dementia and comorbid SMI were also more likely to be younger [t(20826) = 26.16; p<0.001], from a minority ethnic group [χ^2^ (4) = 127.31; p <0.001], not married [χ^2^(1) = 63.48; p<0.001], have higher education [χ^2^(1) = 248.42; p <0.001], be more socially deprived [χ^2^(4) = 51.89; p <0.001], at higher CVD risk [χ^2^ (1) = 27.21; p <0.001], currently smoking [χ^2^ (2) = 147.04; p <0.001] and taking antipsychotics [χ^2^(1) = 697.25; p <0.001] and antidepressants [χ^2^(1) = 106.56; p <0.001] (and less likely to be taking dementia-treatment agents [χ^2^(1) = 21.33; p <0.001]) when compared with those without comorbid SMI at first MMSE recorded. Within the group of individuals with dementia and SMI (Table 2), we examined whether there were differences related to the SMI diagnoses (bipolar versus schizophrenia) and we found that individuals with dementia and bipolar disorders had slightly higher MMSE scores than those individuals with dementia and schizophrenia [t(2640) = −1.39; p <0.001] and those with bipolar were disorders were more likely to be younger [t(2640) = 3.44; p <0.001]. Individuals with schizophrenia were more likely to be from a minority ethnic group [χ^2^ (4) = 63.52; p <0.001], single or separated [χ^2^(1) = 25.27; p <0.001], have lower levels of education [χ^2^(1) = 3.05; p = 0.081], higher levels of social deprivation [χ^2^(4) = 18.26; p < 0.001] and less likely to be taking antidepressant medication [χ^2^(1) = 4.71; p = 0.029] compared to those with bipolar disorders. There were no significant differences for gender, first language, smoking, CVD risk and other medication intake. Fisher exact Chi-square tests were additionally performed to explore the potential association between CVD risk and antipsychotic and no significant associations were found (p = 0.33 and p = 0.79, respectively).

**TABLE 1:** Descriptive Statistics at Baseline for Maximal Sample Available (N = 4718) and Individuals With Dementia Alone and Individuals With Comorbid SMI

	Total Sample (N = 4,718)		Dementia Alone (n = 4,162)		Dementia and SMI (n = 556)	
	n	Mean (SD)	n	Mean (SD)	N	Mean (SD)
MMSE***	4718	19.8(6.2)	4162	19.7(6.2)	556	20.8(6.3)
Age at Baseline***	4718	77(7.3)	4162	77(6.8)	556	72(8.3)
	n	%	n	%	N	%
Gender						
Female	2878	61	2552	61.3	326	58.6
Male	1840	39	1610	38.7	230	41.4
Ethnicity***						
White	3399	72	3075	73.9	324	58.3
Black African	155	3	127	3.1	28	5
Black Caribbean	712	15	573	13.8	139	25
Indian	176	4	156	3.7	20	3.6
Mixed/Other	255	5	211	5.1	44	7.9
Unknown	21		20	0.5	1	0.2
Marital Status***						
Single/Separated	2905	62.0	2480	59.6	425	76.4
Married/Cohabiting	1762	37.0	1633	39.2	129	23.2
Unknown	51	1	49	1.2	2	0.4
Education***						
Gcse+	1629	35	1308	31.4	321	57.7
Primary/Lower	3089	65	2854	68.6	235	42.3
Language						
English	2544	54	2178	52.3	366	65.8
Not English	179	4	157	3.8	22	4.0
Unknown	1995	42	1827	43.9	168	30.2
IMD***						
[0–10]	431	9.0	412	9.9	19	3.4
[10–20]	785	17.0	715	17.2	70	12.6
[20–30]	1038	22.0	893	21.5	145	26.1
[30–40]	1027	22.0	895	21.5	132	23.7
[40–60]	461	10.0	392	9.4	69	12.4
Unknown	976	21.0	855	20.5	121	21.8
Smoking***						
No	2429	51.0	2195	52.7	234	42.1
Current	1303	28.0	1041	25.0	262	47.1
Past	494	10.0	460	11.1	34	6.1
Unknown	492	10.0	466	11.2	26	4.7
CVD Risk***						
Normal	586	12.0	532	12.8	54	9.7
At Risk	2599	55.0	2174	52.2	425	76.4
Unknown	1533	32.0	1456	35.0	77	13.8
Antidementia***						
No	4453	96.9	3904	96.7	549	99.2
Yes	265	3.1	258	3.3	7	0.8
Antipsychotics***						
No	4596	99.4	4134	99.9	462	93.7
Yes	122	0.6	28	0.1	94	6.3
Antidepressant***						
No	4625	99.6	4112	99.8	513	97.4
Yes	93	0.4	50	0.2	43	2.6

GCSE+: qualification at least General Certificate of Secondary Education, or equivalent; IMD: index of multiple deprivation.

⁎⁎⁎ p <.05.

**TABLE 2:** Descriptive Statistics at Baseline for Sample of Individuals With Dementia and SMI (N = 556)

	Dementia and Bipolar (n = 124)		Dementia and Schizophrenia (n = 432)	
	N	Mean (SD)	N	Mean (SD)
MMSE^c^	124	21.4 (6.1)	432	20.7 (6.1)
Age at Baseline^c^	124	70.3 (7.5)	432	72.1 (8.3)
	**N**	**%**	**N**	**%**
Gender				
Female	64	51.9	262	60.6
Male	60	48.1	170	39.4
Ethnicity^c^				
White	95	76.7	229	53.0
Black African	5	3.8	23	5.3
Black Caribbean	8	6.9	131	30.3
Indian	8	6.2	12	2.8
Mixed/Other	8	6.4	36	8.3
Unknown	0	0	1	0.2
Marital Status^c^				
Single/Separated	82	66.4	343	79.4
Married/Cohabiting	42	33.6	87	20.1
Unknown	0	0	2	0.5
Education^a^				
GCSE+	83	67.2	238	55.1
Primary/Lower	41	32.8	194	44.9
Language				
English	78	62.6	288	66.7
Not English	8	6.7	14	3.2
Unknown	38	30.6	130	30.1
IMD^b^				
[0–10]	10	7.7	9	2.1
[10–20]	28	22.9	42	9.7
[20–30]	30	24.1	115	26.6
[30–40]	15	11.9	117	27.1
[40–60]	18	14.9	51	11.8
Unknown	23	18.4	98	22.7
Smoking				
No	65	52.7	169	39.1
Current	51	40.8	211	48.8
Past	5	4.2	29	6.7
Unknown	3	2.3	23	5.3
CVD Risk				
Normal	11	8.6	43	10.0
At Risk	98	79	327	75.7
Unknown	15	12.4	62	14.4
Antidementia				
No	122	98.4	425	98.8
Yes	2	1.6	5	1.2
Antipsychotics				
No	113	91.3	349	80.8
Yes	11	8.7	83	19.2
Antidepressant^a^				
No	117	94.2	396	91.7
Yes	7	5.8	36	8.3

IMD: index of multiple deprivation; GCSE+: qualification at least General Certificate of Secondary Education, or equivalent.

a p <0.05.

b p <0.01.

c p <0.001 from exact chi-square and T-tests between groups.

### Linear Mixed Models

**FIGURE 1:**
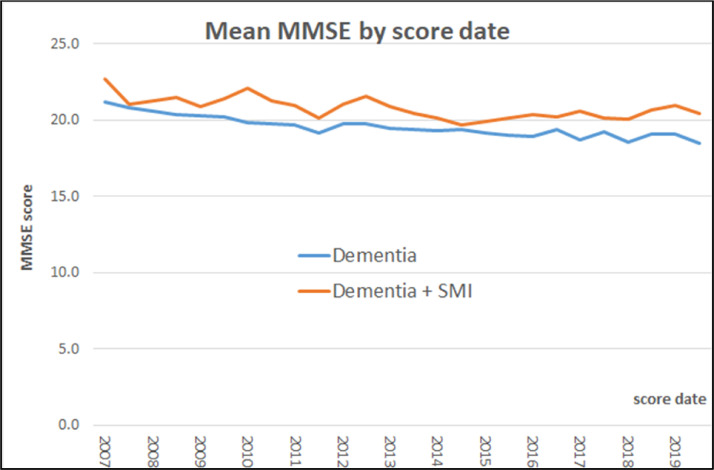
MMSE means over time in individuals with dementia with and without comorbid SMI.

When we investigated MMSE trajectories for this sample, we found that linear models showed a better fit than quadratic models (linear BIC 107342 versus quadratic BIC: 108301). The ICC for the unconditional model was 0.75, indicating significant between- and within-individual variability. At intercept level (Table 3), we found that individuals with dementia and SMI had higher MMSE scores than those with dementia alone. Lower MMSE scores were found in individuals from a minority background (Black African and Caribbean), those with lower levels of education and those whose first language was not English, and with higher levels of social deprivation. Current smokers, individuals classified at CVD risk or those to be taking dementia or antipsychotic medication were also found to have lower MMSE scores when compared with those that never smoked, were not classified at CVD risk and not taking dementia or antipsychotic medication. At slope level (Table 4 and Fig. 1), we found that although individuals with dementia and SMI showed an accelerated decline in their MMSE scores when compared to those with only dementia in unadjusted (model 1 in Table 4). Effect sizes for slopes were of 0.20 for differences between dementia only and dementia with comorbid SMI. These estimates are common in these studies and considered meaningful.^27^ When adjustments were considered (model 2, 3, and 4 in Table 4); this association was partially attenuated when models were additionally adjusted by socio-demographics, smoking, CVD risk factors and further attenuated when adjusted by medication (model 5 in Table 4). When the association of each specific type of medication was examined, our results showed that this attenuation is associated with dementia, antipsychotics, and antidepressants independently (models 5a, 5b, and 5c in Table 4).

**TABLE 3:** Estimates for the Fully Adjusted Linear Mixed Model for MMSE Trajectories (n = 4,718, 20,828 Observations)

	Intercept		Slope	
	Estimate (SE)	CI 95%	Estimate (SE)	CI 95%
Intercept	26.21 (1.45)^c^	[23.369, 29.052]		
Slope linear			−0.377 (0.235)	[−0.837, 0.083]
Diagnoses				
Dementia + SMI	0.97 (0.389)^a^	[0.208, 1.732]	−0.133 (0.061)^a^	[−0.252, −0.014]
Age	0.019 (0.018)	[−0.016, 0.053]	0.01 (0.003)^c^	[0.004, 0.015]
Gender (ref: Female)				
Male	0.253 (0.259)	[−0.254, 0.76]	−0.037 (0.042)	[−0.119, 0.044]
Ethnicity (ref: White)				
Black African	−2.732 (0.736)^c^	[−4.175, −1.289]	−0.285 (0.114)^a^	[−0.509, −0.062]
Black Caribbean	−1.788 (0.351)^c^	[−2.476, −1.1]	−0.104 (0.055).	[−0.212, 0.005]
Indian	−0.024 (0.664)	[−1.325, 1.277]	−0.134 (0.101)	[−0.332, 0.063]
Mixed/other	−0.156 (0.577)	[−1.287, 0.975]	−0.097 (0.087)	[−0.268, 0.074]
Unknown	−1.227 (3.431)	[−7.951, 5.498]	−0.243 (0.398)	[−1.023, 0.536]
Marital status (ref: Married)				
Single or separated	−0.465 (0.265).	[−0.984, 0.054]	−0.066 (0.043)	[−0.15, 0.018]
Unknown	−3.585 (1.536)^a^	[−6.596, −0.575]	−0.668 (0.212)^b^	[−1.084, −0.252]
Education (ref: GSCE+)				
Primary or lower	−0.916 (0.261)^c^	[−1.429, −0.403]	−0.03 (0.042)	[−0.113, 0.052]
First language (ref: English)				
Not English	−3.962 (0.637)^c^	[−5.212, −2.713]	−0.148 (0.105)	[−0.354, 0.057]
Unknown	0.483 (0.255).	[−0.016, 0.981]	−0.029 (0.041)	[−0.109, 0.052]
IMD (ref: [0–10])				
[10–20]	−0.3 (0.482)	[−1.245, 0.646]	0.1 (0.08)	[−0.057, 0.257]
[20–30]	−0.823 (0.464).	[−1.732, 0.085]	0.071 (0.077)	[−0.08, 0.223]
[30–40]	−1.495 (0.47)^b^	[−2.417, −0.574]	0.039 (0.078)	[−0.114, 0.193]
[40–60]	−1.316 (0.556)^a^	[−2.406, −0.226]	0.061 (0.091)	[−0.117, 0.239]
Unknown	−2.079 (0.472)^c^	[−3.005, −1.154]	−0.027 (0.079)	[−0.181, 0.128]
Smoking (ref: No)				
Current	−1.018 (0.287)^c^	[−1.582, −0.455]	−0.11 (0.046)^a^	[−0.201, −0.019]
Former	0.34 (0.422)	[−0.486, 1.167]	0.029 (0.067)	[−0.103, 0.161]
Unknown	−1.167 (0.404)^b^	[−1.96, −0.375]	0.059 (0.073)	[−0.083, 0.201]
CVD risk (ref: No)				
Yes	−2.067 (0.409)^c^	[−2.868, −1.266]	−0.382 (0.068)^c^	[−0.515, −0.249]
Unknown	−0.738 (0.378).	[−1.478, 0.003]	−0.136 (0.062)^a^	[−0.257, −0.014]
Antidementia	−2.163 (0.446)^c^	[−3.037, −1.289]	0.439 (0.086)^c^	[0.271, 0.606]
Antipsychotic	−3.39 (0.745)^c^	[−4.849, −1.931]	−0.275 (0.127)^a^	[−0.523, −0.026]
Antidepressant	1.437 (0.79)^a^	[−0.11, 2.985]	0.274 (0.144).	[−0.008, 0.555]
Variances				
Intercept	28.816 (5.368)			
Slope	0.609 (0.781)			
Residual	11.601 (3.406)			
Fit statistics for linear models				
BIC	123788.715			
-2LL	**−**61625.868			

a p <0.05.

b p <0.01.

c p <0.001.

**TABLE 4:** Slope Estimates for Individuals With Dementia and SMI (Reference Category: Only Dementia) for MMSE Trajectories Unadjusted and Adjusted Models (n = 4,718, 20,828 Observations)

	M (SE)	95% CI
Model 1: unadjusted	−0.186 (0.056)^c^	[−0.297, −0.076]
Model 2: adjusted by socio-demographics	−0.167 (0.058)^b^	[−0.28, −0.053]
Model 3: additionally adjusted by smoking	−0.155 (0.058)^b^	[−0.269, −0.041]
Model 4: additionally adjusted by CVD risk	−0.153 (0.058)^b^	[−0.298, −0.069]
Model 5: additionally adjusted by medication	−0.133 (0.061)^a^	[−0.252, −0.014]
Models with adjustments for medication groups		
Model 5a: Model 4 with only dementia	−0.152 (0.058)^b^	[−0.286, −0.057]
Model 5b: Model 4 with only antipsychotic	−0.144 (0.061)^a^	[−0.264, −0.025]
Model 5c: Model 4 with only antidepressant	−0.19 (0.059)^b^	[−0.306, −0.075]

a p <0.05.

b p <0.01.

c p <0.001.

When we further investigated whether different SMI diagnosis (schizophrenia and bipolar disorders) showed different cognitive trajectories when compared with individuals with only dementia (Table 5), we found that, at intercept level, individuals with schizophrenia and bipolar disorders had higher MMSE scores at intercept level when compared with individuals without comorbid SMI, even after adjusting for relevant covariates. However, post hoc analyses did not reveal significant differences between those with schizophrenia and bipolar disorders at this level. At slope level (Table 5), our results showed differences between the trajectories of individuals with schizophrenia and bipolar disorders, before and after relevant adjustments. An acceleration in cognitive decline was found in individuals with bipolar disorders compared to those with only dementia, while the trajectories with schizophrenia did not seem to be different from those that had dementia without comorbid SMI. Post hoc analyses confirmed the differences between the trajectories of those with bipolar disorders and those with schizophrenia and effect sizes for slopes were equal to 0.17 (although relatively moderate these can still be considered meaningful^27^).

**Table 5:** Intercept and Slope Estimates for Individuals With Dementia and SMI (Split into Bipolar and Schizophrenia) (Reference Category: Only Dementia) for MMSE Trajectories Unadjusted and Adjusted Models *(*n = 4,718, 20,828 Observations)

Intercept for Fully Adjusted Model	M (SE)	95% CI
Diagnoses (ref: Dementia only)		
Dementia + bipolar	0.871 (0.801)^a^	[−0.699, 2.442]
Dementia + schizophrenia	2.337 (0.478)^c^	[1.4, 3.274]
Slope		
	M (SE)	95% CI
Model 1: unadjusted		
Dementia + bipolar	−0.343 (0.121)^b^	[−0.58, −0.107]
Dementia + schizophrenia	−0.036 (0.071)	[−0.175, 0.102]
Model 2: adjusted by socio-demographics		
Dementia + bipolar	−0.357 (0.12)^b^	[−0.593, −0.121]
Dementia + schizophrenia	−0.005 (0.072)	[−0.146, 0.135]
Model 3: additionally adjusted by smoking		
Dementia + bipolar	−0.342 (0.121)^b^	[−0.578, −0.106]
Dementia + schizophrenia	0.014 (0.072)	[−0.127, 0.155]
Model 4: additionally adjusted by CVD risk		
Dementia + bipolar	−0.361 (0.12)^b^	[−0.597, −0.126]
Dementia + schizophrenia	−0.012 (0.072)	[−0.153, 0.13]
Model 5: additionally adjusted by medication		
Dementia + bipolar	−0.296 (0.121)^a^	[−0.534, −0.059]
Dementia + schizophrenia	−0.063 (0.075)	[−0.084, 0.209]
Models with adjustments for medication groups		
Model 5a: Model 4 with only antidementia		
Dementia + bipolar	−0.347 (0.119)^b^	[−0.58, −0.114]
Dementia + schizophrenia	0.0 (0.071)	[−0.14, 0.14]
Model 5b: Model 4 with only antipsychotic		
Dementia + bipolar	−0.296 (0.123)^a^	[−0.536, −0.056]
Dementia + schizophrenia	−0.047 (0.075)	[−0.101, 0.194]
Model 5c: Model 4 with only antidepressant		
Dementia + bipolar	−0.377 (0.121)^b^	[−0.615, −0.14]
Dementia + schizophrenia	−0.015 (0.072)	[−0.157, 0.127]

a p <0.05.

b p <0.01.

c p <0.001.

When sensitivity analyses included age at diagnoses similar trends were found. When we compared results with only complete cases for all covariates (n = 1453) and only those individuals that were still alive in 2019 (n = 1766) we found similar trends as slope estimates were reduced but in the same direction for fully adjusted models even considering the different length of follow up between both groups (individuals with dementia and SMI had longer follow ups (M = 4.7; SD = 2.7) compared to those that had only dementia (M = 3.1; SD = 2.01).

## Discussion

This study examined trajectories of cognitive performance in individuals diagnosed with dementia, comparing those with and without a comorbid SMI diagnosis. This study used data from a large longitudinal mental healthcare case register, and considered sociodemographic factors, health behaviors, cardiovascular risk factors, and medication as covariates.

Our primary finding was that individuals with dementia with SMI diagnoses had a faster cognitive decline when compared with those without comorbid SMI. However, this association was partially explained by socio-demographic, health behaviors and CVD risk factors; and more substantially attenuated when medication was considered. Associations of socio-demographic and health-related factors with cognitive trajectories are broadly consistent with previous research in British ageing cohorts.^28^ Higher educated individuals had higher intercept coefficients, in line with cognitive reserve hypotheses,^29^ but no difference in rate of decline which is consistent with findings of Davis et al.^28^ Moreover, individuals who were current smokers and showed higher number of CVD risk factors were more likely to have a faster decline as found in previous studies.^28^ People with SMI are also known to be more likely to be smokers and have higher CVD risk.^30^ Given that these factors accounted for a reasonable proportion of the overall association between SMI and faster decline, future research on interventions in this population should explore further the beneficial cognitive effects of smoking cessation and physical activity interventions and whether public health strategies for dementia prevention could be adapted for individuals with SMI. Given the impact of health behaviors and CVD risk factors on cognitive trajectories, especially in older adults, it is important that our health care pathway addressed these at the onset of the illness, bringing primary/general medical care into the psychiatric clinic early on.

When we explored whether there were differences between those individuals with schizophrenia and bipolar disorders compared to those with dementia without comorbid SMI, we found that there those with dementia with comorbid SMI had higher MMSE scores at intercept level, even after adjusting for age. However, no differences were found between those individuals with schizophrenia and bipolar disorders. Although our findings are not directly comparable with previous research in individuals without dementia, they are consistent with previous cross-sectional studies which found similar profiles.^2^^,^^3^ Furthermore, the differences found at slope level in our study provides some evidence in line with those studies that suggested that differences might be arise when exploring this association in longitudinal settings^4^ but differs from those that found no differences between cognitive trajectories in individuals with schizophrenia and bipolar disorders.^5^, ^6^, ^7^ This could be associated to the fact that previous research had smaller sample sizes and they might have been underpowered to detect subtle cognitive changes over time or to reveal differences between disorders^1^ or the fact that our study, and therefore, cohort is restricted to individuals with comorbid dementia. Future research should explore whether these differences might be associated to the different underlying biological mechanisms of schizophrenia and bipolar disorders or with the potentially shared mechanisms between disorders and dementia.

With regards to medication, adjustment for dementia, antipsychotic, and antidepressant medications strongly impacted the association of interest even after accounting for the other covariates considered such as CVD risk. When we examined the role of each medication independently, we found that this impact was clear for dementia and antidepressants, the estimates suggest that there could be a greater and potentially protective impact for antipsychotic medication in the case of bipolar disorders.

Besides the strengths of our study associated with the large sample from a traditionally underrepresented population in cognitive decline studies and the richness of the longitudinal nature of the data, some limitations should be acknowledged. First, data on health behaviors was limited to smoking status, and we could not include important potential predictors such as alcohol consumption or physical activity in our models. Future studies are therefore needed to understand further the role of lifestyle and health when understanding cognitive decline in individuals with these comorbidities. Second, dementia was ascertained using routine clinical diagnoses rather than research instruments and we did not explore differences between diagnostic subgroups due to their limited sample sizes and the difficulty to distinguish those clinical diagnoses that are often recorded to facilitate and speed the patient´s journey through the different healthcare pathways. Third, there could be some potential selection bias associated to the fact that: our sample was limited to those with at least three MMSE scores available (following methodological guidelines), dementia can go undiagnosed in SMI and our population has an increased risk of premature mortality.^31^ Although the direction of our results were robust when performing sensitivity analyses restricted to only those that were still alive at the end of the study period, the attenuation of the estimate suggests that individuals closer to their death were more likely to exhibit an accelerated decline. These findings are consistent with the terminal cognitive decline hypotheses^32^^,^^33^ and future research should explore further the potential drivers of mortality in this population. Finally, this data only had a global measure of cognition such as MMSE which is well known and used for comparative purposes; however, some research has highlighted that there could be a different impact for different cognitive domains as a function of SMI.^34^

To conclude, our findings showed that individuals with comorbid SMI show a faster cognitive decline when compared with those that have dementia without comorbid SMI. This accelerated decline seems to be more evident in individuals with bipolar disorders when compared with individuals with dementia only and it is only partially explained by relevant confounders. Further research is needed to detangle the biological explanatory pathways. Clinical and public health interventions should monitor medication regimes and promote healthy lifestyles in individuals diagnosed with dementia and SMI to reduce the potential risk of accelerated cognitive decline in this population.

## Authors contributions

RB conceived the idea, designed the study and the analytical plan. AM extracted, preprocessed, and analyzed the data. RS provided clinical input and all authors contributed to the interpretation of the results. RB drafted the manuscript and all authors made substantial contributions, revised this work critically and approved final version to be published.

RB is funded in part by grant MR/R016372/1 for the King's College London MRC Skills Development Fellowship programme funded by the UK Medical Research Council (MRC, https://mrc.ukri.org) and by grant IS-BRC-1215-20018 for the National Institute for Health Research (NIHR, https://www.nihr.ac.uk) Biomedical Research Centre at South London and Maudsley NHS Foundation Trust and King's College London. AM is funded by Takeda California, Inc. RD, RS, AR are part-funded by the National Institute for Health Research (NIHR) Biomedical Research Centre at South London and Maudsley NHS Foundation Trust and King's College London. RD is supported by: (1) NIHR Biomedical Research Centre at South London and Maudsley NHS Foundation Trust and King's College London, London, U.K. (2) Health Data Research UK, which is funded by the UK Medical Research Council, Engineering and Physical Sciences Research Council, Economic and Social Research Council, Department of Health and Social Care (England), Chief Scientist Office of the Scottish Government Health and Social Care Directorates, Health and Social Care Research and Development Division (Welsh Government), Public Health Agency (Northern Ireland), British Heart Foundation and Wellcome Trust. (3) The BigData@Heart Consortijum, funded by the Innovative Medicines Initiative-2 Joint Undertaking under grant agreement No. 116074. This Joint Undertaking receives support from the European Union's Horizon 2020 research and innovation programme and EFPIA; it is chaired by DE Grobbee and SD Anker, partnering with 20 academic and industry partners and ESC. (4) The National Institute for Health Research University College London Hospitals Biomedical Research Centre. (5) National Institute for Health Research (NIHR) Biomedical Research Centre at South London and Maudsley NHS Foundation Trust and King's College London. (5) The UK Research and Innovation London Medical Imaging & Artificial Intelligence Centre for Value Based Healthcare. (6) National Institute for Health Research (NIHR) Applied Research Collaboration South London (NIHR ARC South London) at King's College Hospital NHS Foundation Trust.

This paper represents independent research part-funded by the National Institute for Health Research (NIHR) Biomedical Research Centre at South London and Maudsley NHS Foundation Trust and King's College London. The views expressed are those of the author(s) and not necessarily those of the NHS, the NIHR or the Department of Health and Social Care. The funders had no role in study design, data collection and analysis, decision to publish, or preparation of the manuscript.

The authors report no conflicts with any product mentioned or concept discussed in this article.

## Appendix SUPPLEMENTARY MATERIALS

Image, application 1

## Footnotes

Supplementary material associated with this article can be found, in the online version, at https://doi.org/10.1016/j.jagp.2020.10.018.
