# Supplementary material for: Cognitive Trajectories in Comorbid Dementia With Schizophrenia or Bipolar Disorder: The South London and Maudsley NHS Foundation Trust Biomedical Research Centre (SLaM BRC) Case Register
Source: Am J Geriatr Psychiatry. 2021 Jun;29(6):604–16. doi: 10.1016/j.jagp.2020.10.018 (PMC8169045; doi:10.1016/j.jagp.2020.10.018)
Supplement: Supplementary file 1 [file mmc1.pdf]

Supplemental Online Appendix

**Antipsychotics**	**Antidepressants**	**Antidementia**
Abilify	Agomelatine	Donepezil
Amisulpride	Alventa	Galantamine
Aripiprazole	Amitriptyline	Memantine
Atrolak	Amphero	
Benperidol	Anafranil	
Biquelle	Brintellix	
Brancico	Cipralex	
Chlorpromazine	Cipramil	
Clopixol	Citalopram	
Clopixol Acuphase	Citalopram Hydrobromide	
Clozapine	Citalopram Hydrochloride	
Clozaril	Clomipramine	
Denzapine	Cymbalta	
Depixol	Depefex	
Depixol-Conc	Dosulepin	
Dolmatil	Doxepin	
DROPERIDOL	Duciltia	
Ebesque	Duloxetine	
Fentazin	Edronax	
Flupentixol	Efexor	
Flupentixol Decanoate	Escitalopram	
Flupentixol Hydrochloride	Faverin	
Fluphenazine	Fluanxol	
Fluphenazine Decanoate	Fluoxetine	
Fluphenazine Hydrochloride	Flupentixol	
Haldol	Flupentixol Hydrochloride	
Haloperidol	Fluvoxamine	
Haloperidol Decanoate	Imipramine	
Invega	Isocarboxazid	
Largactil	Lofepramine	
Latuda	Lustral	
Levinan	Manerix	
Levomeprom	Maprotiline	
Levomepromazine	Mianserin	
Levomepromazine Hydrochloride	Mirtazapine	
Levomepromazine Maleate	Moclobemide	
Lurasidone	Molipaxin	
Melleril	Nardil	
Melperone	Nefazodone	
Mintreleq	Nortriptyline	
Modecate	Olena	
Nozinan	Optimax	
Olanzapine	Oxactin	

Orap	Oxitriptan	
Paliperidone	Paroxetine	
PENFLURIDOL	Phenelzine	
Pericyazine	Politid	
Perphenazine	Prothiaden	
Pimozide	Prozac	
Piportil	Prozep	
Piportil Depot	Reboxetine	
Pipothiazine	Rodomel	
Pipothiazine Palmitate	Seroxat	
Pipotiazine	Sertraline	
PIPOTIAZINE PALMITATE	Sunveniz	
Promazine	Surmontil	
Psyquet	Tonpular	
Psytixol	Tranylcypromine	
Quetiapine	Trazodone	
Risperdal	Trimipramine	
Risperdal Consta	Triptafen	
Risperidone	Tryptophan	
Seotiapim	Valdoxan	
Serenace	Venaxx	
Seroquel	Vencarm	
Solian	Venlablue	
Sondate	Venladex	
Sulpiride	Venlafaxine	
Tenprolide	Venlalic	
Thioridazine	Venlaneo	
Trevicta	Vensir	
Trifluoperazine	ViePax	
Veractil	Vortioxetine	
Xeplion	Winfex	
Zalasta	Zispin	
Zaluron		
Zaponex		
Ziprasidone		
Ziprasidone Hydrochloride		
Zuclopenthixol		
Zuclopenthixol Acetate		
Zuclopenthixol Decanoate		
Zuclopenthixol Hydrochloride		
Zypadhera		
Zyprexa		
